# Anti-inflammatory effect of curcumin on neurological disorders: a narrative review

**DOI:** 10.3389/fphar.2025.1658115

**Published:** 2025-10-08

**Authors:** Baoyin Zhou, Binbin Hu

**Affiliations:** ^1^ Queen Mary School, Jiangxi Medical College, Nanchang University, Nanchang, China; ^2^ Department of Neurology, The Second Affilliated Hospital of Nanchang University, Nanchang University, Nanchang, China

**Keywords:** curcumin, neuroinflammation, neurological disorders, reactive oxygen species, neuroprotection

## Abstract

Neuroinflammation arises from the synergistic interplay of multiple inflammatory mediators and is pathologically associated with various neurological disorders. These conditions are complex, multifactorial diseases characterized by dynamic interactions between chronic neuroinflammation, oxidative stress, and progressive neuronal degeneration. Curcumin, a naturally occurring polyphenolic compound, exhibits significant pharmacological activity in anti-inflammatory processes and immune regulation. Within neuroinflammatory pathologies, microglial cells are crucial effector cells as they can secrete inflammatory mediators. Emerging evidence suggests that these resident immune cells are the primary site of the biological activity of curcumin in the central nervous system. The compound demonstrates multimodal regulatory effects, including modulation of key signaling pathways (NF-κB, NLRP3 inflammasome, and Nrf2) and upregulation of anti-inflammatory cytokines (TGF-β and interleukin-10), collectively contributing to the neuroinflammatory suppression effect of curcumin. This review comprehensively analyzed the therapeutic potential of curcumin in neuroinflammation and explored its clinical prospects for neurological disease intervention.

## 1 Introduction

Neuroinflammation is a sophisticated immunological response within the central nervous system that is pivotal in neuroprotective and neurodegenerative processes (Botella Lucena and Heneka, 2024; Lei et al., 2025; Pluta, 2025). This phenomenon is characterized by the activation of microglia and astrocytes, accompanied by the release of pro-inflammatory cytokines, including tumor necrosis factor-α (TNF-α), interleukin-1β (IL-1β), and interleukin-6 (IL-6), and reactive oxygen species (ROS) (Chen et al., 2024; Du et al., 2024; Kong et al., 2024b; Liu et al., 2024c). Acute neuroinflammation serves essential functions in tissue repair and pathogen clearance. However, chronic neuroinflammatory responses contribute significantly to the pathogenesis of various neurological disorders, such as Alzheimer’s disease (AD) (Chae et al., 2024), Parkinson’s disease (PD) (Qi et al., 2025), multiple sclerosis (MS) (Woo et al., 2024), and stroke (Guan et al., 2024). The underlying mechanisms involve persistent microglial activation mediated by pattern recognition receptors, including Toll-like receptors (TLRs) and the NLRP3 inflammasome, leading to neuronal damage through excessive cytokine production and oxidative stress (Zhang et al., 2021; Hou et al., 2024; Xu et al., 2025a). Furthermore, disruption of the blood-brain barrier facilitates the infiltration of peripheral immune cells, exacerbating neuroinflammatory conditions (Takata et al., 2021; Hou et al., 2023). Emerging therapeutic approaches targeting neuroinflammatory pathways, particularly the inhibition of NF-κB or NLRP3 inflammasome signaling, have demonstrated promising results in preclinical studies (Lei et al., 2023; Zhang et al., 2023a). Given the limitations of conventional anti-inflammatory medications, including adverse effects associated with non-steroidal anti-inflammatory drugs and corticosteroids, researchers are showing increasing interest in identifying natural compounds that exhibit potent anti-inflammatory properties while maintaining favorable safety profiles. This shift in therapeutic focus reflects the need for more targeted and tolerable interventions in neuroinflammatory disorders.

**FIGURE 1:**
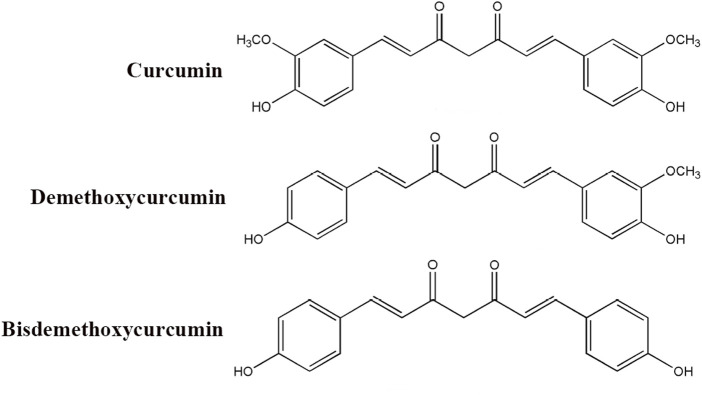
Chemical structure of curcuminoids (curcumin, demethoxycurcumin, and bisdemethoxycurcumin).

Curcumin, a naturally occurring polyphenolic compound derived from the rhizomes of *Curcuma longa*, has garnered significant attention in biomedical research owing to its diverse pharmacological properties encompassing anti-inflammatory, antioxidant, anticancer, and neuroprotective activities (Jabczyk et al., 2021; Ran et al., 2021; Liu et al., 2023a; Sadeghi et al., 2023; Wang et al., 2023). Curcumin, demethoxycurcumin, and bisdemethoxycurcumin are called curcuminoids (Figure 1). As the principal bioactive constituent of turmeric, curcumin has been extensively investigated for its capacity to modulate multiple signaling pathways, including NF-κB, mitogen-activated protein kinase, and phosphatidylinositol 3-kinase/Akt (PI3K/Akt) cascades, which play pivotal roles in regulating inflammatory responses, cellular proliferation, and apoptotic processes (Qiu et al., 2020; Ren et al., 2020; Ran et al., 2021; Shamsnia et al., 2023). Despite its considerable therapeutic potential, the clinical application of curcumin has been constrained by pharmacokinetic limitations, particularly its poor aqueous solubility, rapid metabolic degradation, and systemic elimination. Therefore, contemporary research has focused on developing advanced drug delivery platforms, including nanoparticle formulations, liposomal carriers, and phospholipid complexes, to enhance the bioavailability and pharmacokinetic profile of curcumin (Chen et al., 2020; Quispe et al., 2021; Hassanizadeh et al., 2023; Mohammadzadeh et al., 2024). Emerging scientific evidence further suggests that the therapeutic effects of curcumin are mediated through epigenetic regulatory mechanisms, including modulation of histone acetylation patterns and DNA methylation status, which potentially contribute to its chemopreventive and antineoplastic properties (Fabianowska-Majewska et al., 2021; Yang et al., 2021b; Ming et al., 2022; Sawesi et al., 2022). Current reviews on curcumin are focused on elucidating its therapeutic targets in neurological disorders (Zia et al., 2021; Sadeghi et al., 2023; Nunes et al., 2024; Yang et al., 2024a). Although its interactions with numerous molecular targets have been extensively documented, there remains a lack of comprehensive review addressing the specific mechanisms through which curcumin modulates inflammation in the treatment of neurological disorders (Menon and Sudheer, 2007; Peng et al., 2021; Dehzad et al., 2023; Sadeghi et al., 2023). A deeper understanding of anti-inflammatory mechanisms is crucial for the development of curcumin-based therapeutic strategies and their integration into routine clinical practice.

In summary, curcumin has remained a subject of significant scientific interest due to its long history in traditional medicine and its considerable potential in modern biomedical research. Its complex chemical structure, broad pharmacological activities, and challenges related to bioavailability offer compelling avenues for ongoing and future investigations. As research continues to elucidate its mechanisms of action at the molecular level and to improve its delivery methods, curcumin holds promise as a key agent in the management of various health conditions. This review provides a comprehensive exploration of the specific mechanisms by which curcumin treats neurological diseases through the regulation of inflammation. It systematically examines the role of curcumin in mitigating neuroinflammation via the gut–brain axis, outlining how it indirectly ameliorates neuroinflammatory processes by modulating the gut microbiota, maintaining intestinal barrier integrity, and subsequently reducing systemic inflammation. Furthermore, the review offers an in-depth analysis of novel materials (e.g., nanomaterials) developed to enhance its bioavailability—particularly within the context of neurological therapy—and evaluates innovative strategies currently under development aimed at optimizing its clinical efficacy.

## 2 Synthesis and metabolism of curcumin

Curcumin (1,7-bis(4-hydroxy-3-methoxyphenyl)-1,6-heptadiene-3,5-dione), is a naturally occurring polyphenolic compound that serves as the principal bioactive constituent extracted from the rhizomes of *Curcuma longa*, a perennial herbaceous plant belonging to the Zingiberaceae family (Liu et al., 2023b; Wang et al., 2023). For centuries, it has been traditionally employed for medicinal purposes, culinary applications, and as a coloring agent (Mohamadian et al., 2022; Kuzminska et al., 2024). The ubiquitous presence of curcumin across various plant taxa underscores its ecological significance and therapeutic potential, warranting further investigation into its biosynthetic pathways and health-promoting properties.

## 3 Curcumin inhibits the pro-inflammatory activation of immune cells in the central nervous system

### 3.1 Macrophages

Curcumin exhibits significant immunomodulatory effects on macrophages, which are pivotal innate immune cells involved in inflammatory responses and tissue homeostasis. Experimental studies demonstrate that curcumin preferentially suppresses pro-inflammatory M1 macrophage polarization while promoting anti-inflammatory M2 phenotype, primarily by inhibiting the signal transducer and activator of transcription 3 (STAT3) signaling pathway (Ran et al., 2021; Abdollahi et al., 2023; Deswal et al., 2024). At the molecular level, curcumin reduces lipopolysaccharide (LPS)-induced production of TNF-α, IL-6, and nitric oxide in macrophages by downregulating iNOS and COX-2 expression (Wang et al., 2019). In ischemic stroke models, curcumin administration attenuates stroke-induced white matter damage, improves functional outcomes, and reduces microglial pyroptosis, mediated at least partially through the suppression of NF-κB/NLRP3 signaling pathways (Ran et al., 2021). Similarly, in myocardial infarction, curcumin improves cardiac function and reduces post-MI fibrosis by inhibiting macrophage-fibroblast crosstalk during the acute phase and suppressing IL-18-TGF-β1-p-SMAD2/3 signaling in cardiac fibroblasts (Zhao et al., 2021). In addition, it modulates myocardial inflammation through AMPK-regulated M1/M2 macrophage polarization (Yan et al., 2021). Additional evidence indicates that curcumin protects against particulate matter-induced lung injury by suppressing oxidative stress and inflammatory activation in macrophages (Lee et al., 2023). In ulcerative colitis models characterized by dysregulated M1/M2 macrophage ratios and enhanced M1 activation, curcumin treatment normalizes aberrant macrophage activation, inhibits macrophage chemotaxis, and alleviates inflammatory responses (Chen et al., 2023). Collectively, these findings establish macrophages as crucial cellular mediators of the immunomodulatory actions of curcumin, playing a vital role in regulating immune responses and potentially ameliorating macrophage-associated systemic and neuroinflammatory conditions. The compound demonstrates therapeutic potential across multiple disease states by modulating macrophage polarization and function.

### 3.2 Microglia

Emerging research has elucidated the potent regulatory effects of curcumin on microglia, the resident immune cells of the central nervous system. Under neuroinflammatory conditions, curcumin (5-25 μM) can attenuate LPS-induced microglial activation, achieving approximately 60% reduction in pro-inflammatory cytokine release (TNF-α, IL-1β, IL-6) by suppressing the TLR4/MyD88/NF-κB signaling cascade (Gao et al., 2019; Zhang et al., 2019). The compound exhibits anti-inflammatory and antioxidant properties by inhibiting NOX2-mediated ROS production in activated microglia while upregulating the Nrf2/Heme Oxygenase-1 (HO-1) antioxidant pathway (Duan et al., 2022; Lin et al., 2022; Xu et al., 2025a). In models of traumatic brain injury, curcumin treatment mitigates brain damage, reduces IL-1β and IL-6 levels, promotes microglial polarization toward the M2 phenotype, and downregulates C1ql3 protein expression (Zhang et al., 2023b). Similarly, in subarachnoid hemorrhage, curcumin demonstrates neuroprotective effects by suppressing neuronal ferroptosis through the modulation of Nrf2/HO-1 signaling (Xu et al., 2025b). Notably, curcumin metabolites such as tetrahydrocurcumin retain biological activity in regulating microglial triggering receptor expressed on myeloid cells two signaling, potentially explaining the beneficial effects of the compound despite limited blood-brain barrier permeability (Jiang et al., 2023; Genchi et al., 2024). These findings collectively establish microglia as crucial cellular mediators of the immunomodulatory actions of curcumin, highlighting its therapeutic potential in alleviating microglia-associated neuroinflammatory pathologies through multifaceted mechanisms of action. The ability of the compound to modulate microglial activation states and signaling pathways positions it as a promising candidate for neuroinflammatory intervention.

### 3.3 T cell

**FIGURE 2:**
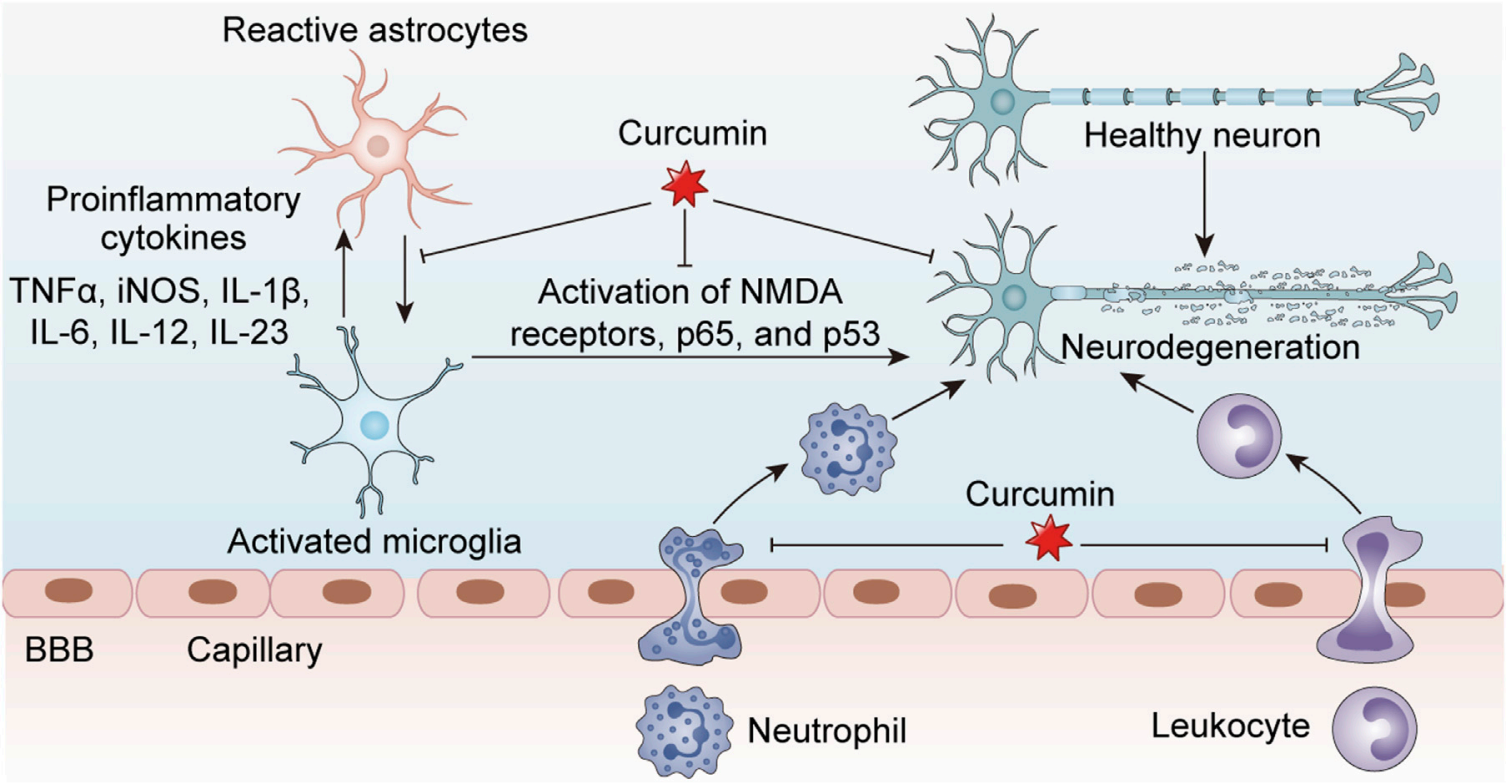
Curcumin exerts inhibitory effects on neuroinflammation associated with central nervous system disorders by modulating the activity of inflammatory cells, including macrophages, microglia, and astrocytes, thereby reducing the release of pro-inflammatory cytokines and subsequently protecting healthy neurons from damage.

Curcumin demonstrates significant immunomodulatory capacity in T cell-mediated diseases through pleiotropic mechanisms targeting cellular activation, differentiation, and effector functions. In autoimmune conditions, curcumin (10–25 μM) ameliorates disease severity by suppressing pathogenic Th1/Th17 responses while enhancing the activity of regulatory T cells (Tregs) (Fasihi et al., 2024; Ghoushi et al., 2024; Nosratabadi et al., 2024). Experimental autoimmune encephalomyelitis (EAE) studies reveal that the neuroprotective effects of curcumin are mediated, at least partially, through AMPK/SIRT1 activation, ultimately attenuating EAE-induced neuronal demyelination, oxidative stress, and neuroinflammation (ELBini-Dhouib et al., 2022; Sadek et al., 2024). Rheumatoid arthritis models demonstrate the ability of curcumin to inhibit Th17 differentiation via suppression of STAT3 phosphorylation, with additional evidence showing its regulation of the inc00052/miR-126-5p/PIAS2 axis through the JAK2/STAT3 signaling pathway (Xiao et al., 2022; Kou et al., 2023; Deng et al., 2024). In allergic disorders, curcumin modulates Th1/Th2 balance by downregulating GATA3 expression and reducing IL-4/IL-5 production in ovalbumin-sensitized models (Wang et al., 2022). Therapeutic applications in thyroid cancer reveal that curcumin can enhance anti-tumor immunity in anaplastic thyroid carcinoma by boosting CD8^+^ T-cell function and inactivating the AKT/mTORC1/STAT3/PD-L1 axis (Vaiss et al., 2024). Furthermore, curcumin reduces severity in acute lung injury/acute respiratory distress syndrome and uncontrolled inflammation by promoting naïve CD4^+^ T-cell differentiation into CD4^+^CD25+FOXP3+ Tregs (Chai et al., 2020). In summary, curcumin exhibits broad therapeutic potential across autoimmune diseases, allergic disorders, viral infections, and cancer immunotherapy through its multifaceted immunomodulatory effects on T-cell subsets, highlighting its value as a versatile immunotherapeutic agent (Figure 2).

## 4 Influence of curcumin on various neurological diseases

### 4.1 Alzheimer’s disease (AD)

AD is a progressive neurodegenerative disorder characterized by cognitive decline, memory impairment, and behavioral disturbances and is the most prevalent cause of dementia worldwide (Scheltens et al., 2021; Jucker and Walker, 2023). The neuropathological hallmarks of AD include the accumulation of extracellular amyloid-β (Aβ) plaques and intracellular neurofibrillary tangles composed of hyperphosphorylated tau protein, which collectively contribute to synaptic dysfunction and neuronal degeneration (Graff-Radford et al., 2021; Serrano-Pozo et al., 2021; Ossenkoppele et al., 2022). Despite extensive research, the precise etiology of AD remains unclear, with proposed involvement of genetic predisposition, environmental factors, and metabolic disturbances. Owing to the growing global aging population and limited availability of disease-modifying therapies, elucidating AD pathogenesis is challenging in contemporary neuroscience research. Given the complex and multifactorial nature of AD pathogenesis, developing effective pharmacological interventions remains a formidable challenge in neurology and drug discovery. Current therapeutic approaches primarily focus on symptomatic management, with acetylcholinesterase inhibitors such as donepezil, rivastigmine, and galantamine enhancing cholinergic neurotransmission to alleviate cognitive deterioration (Se Thoe et al., 2021; Liu et al., 2024a). Furthermore, the N-methyl-D-aspartate receptor antagonist memantine provides partial neuroprotection by modulating glutamatergic excitotoxicity (Vaz and Silvestre, 2020; Pardo-Moreno et al., 2022; Varadharajan et al., 2023). However, these treatments offer merely transient symptomatic stabilization without altering disease progression, thus stimulating increasing interest in natural compounds with multimodal neuroprotective properties. Among these compounds, curcumin, the principal bioactive polyphenol derived from *Curcuma longa* (turmeric), has emerged as a promising candidate for AD intervention, owing to its diverse pharmacological activities targeting multiple pathological pathways implicated in AD progression.

Curcumin exhibits multiple pharmacological properties relevant to AD pathogenesis, including anti-amyloidogenic, anti-tau, antioxidant, and anti-inflammatory effects (Aggarwal and Harikumar, 2009; Azzini et al., 2024). Recent studies demonstrate that during AD progression, impaired adult neurogenesis in the dentate gyrus can be ameliorated by curcumin treatment through modulation of GSK3β/Wnt/β-catenin and CREB/BDNF pathways via PI3K/Akt regulation, reducing apoptosis and improving neurogenesis in AD mouse models (Lou et al., 2024). In AD transgenic mice, curcumin administration downregulates hippocampal expression of HMGB1, RAGE, TLR4, and NF-κB, improving cognitive function by suppressing the HMGB1-RAGE/TLR4-NF-κB inflammatory signaling cascade (Han et al., 2021). As a dietary supplement, curcumin benefits patients with insulin resistance, type 2 diabetes (T2D), and AD by reducing circulating levels of IAPP and GSK-3β while alleviating insulin resistance-related markers, consequently lowering the risk of T2D and AD (Thota et al., 2020). Notably, prophylactic administration of curcumin prior to Aβ deposition demonstrates preventive potential for AD, possibly through facilitating Aβ_42_ clearance from the brain to peripheral circulation (Mei et al., 2020). Additionally, curcumin enhances BDNF-ERK signaling to mitigate AD-associated cognitive deficits (Zhang et al., 2015). Emerging evidence reveals that curcumin treatment significantly alters the composition of gut microbiota in AD mice. The compound undergoes biotransformation by gut microbiota through reduction, demethoxylation, demethylation, and hydroxylation, yielding neuroprotective metabolites (Sun et al., 2020; Leblhuber et al., 2021). These findings suggest a gut-liver-brain axis mediating metabolic and cognitive functions, possibly by reducing fatty acid synthesis, altering cholesterol metabolism, inhibiting hepatic lipogenesis-related pathways, and modulating synaptic plasticity-related pathways in the brain, ultimately suppressing weight gain and improving behavioral and cognitive functions (Pluta et al., 2022; Lamichhane et al., 2024).

The therapeutic efficacy of curcumin in AD is considerably constrained by its limited ability to cross the blood-brain barrier. To address this limitation, researchers have explored combination therapy with ginkgo biloba extract and curcumin, demonstrating the suppression of acetylcholinesterase, caspase-3, hippocampal amyloid-β (Aβ1-42), and phosphorylated tau levels and reduced expression of pro-inflammatory cytokines TNF-α and IL-1β in rat models. This combined treatment also significantly decreased malondialdehyde and modulated reduced glutathione levels (Assi et al., 2023). To enhance the neuroprotective effects of curcumin in AD, advanced delivery systems have been developed. Notably, carrier-free curcumin nanoparticles (CNPs) formed through molecular self-assembly exhibit multivalent binding to Aβ, resulting in superior inhibition of Aβ aggregation. Following intranasal administration, these lipid-based formulations release CNPs and cardiolipin in response to the oxidative microenvironment characteristic of AD. The CNPs modulate microglial polarization (M1→M2) via TLR4/NF-κB pathway inhibition, while simultaneously suppressing Aβ aggregation and enhancing microglial phagocytic clearance of Aβ, thereby overcoming barriers to microglial repolarization (Feng et al., 2024). Intravenous administration of novel brain-targeted nanoparticles (Ce/Zr-MOF@Cur-Lf) facilitates rapid brain entry and ameliorates multiple AD pathological features, including neuronal damage, Aβ deposition, cholinergic system dysfunction, oxidative stress, and neuroinflammation (Yang et al., 2024c). Furthermore, localized delivery of curcumin using human hair keratin/chitosan (C/K) hydrogels may enhance neural regeneration and repair nerve damage, representing an innovative approach for targeted therapy.

Collectively, the existing preclinical evidence from *in vitro* and *in vivo* studies substantiates the neuroprotective efficacy of curcumin in AD pathogenesis, primarily mediated through its anti-neuroinflammatory properties and cognitive-enhancing effects. Nevertheless, substantial research efforts are still warranted to bridge the gap between these promising experimental findings and clinical applications, particularly concerning bioavailability optimization and therapeutic regimen standardization (Sun et al., 2024).

### 4.2 Parkinson’s disease (PD)

PD is a progressive neurodegenerative disorder pathologically characterized by selective degeneration of dopaminergic neurons in the substantia nigra pars compacta and the presence of intraneuronal proteinaceous inclusions known as Lewy bodies, predominantly composed of α-synuclein aggregates (Kalia and Lang, 2015; Bloem et al., 2021). Clinically, PD manifests with cardinal motor symptoms including bradykinesia, resting tremor, rigidity, and postural instability, accompanied by various non-motor features such as cognitive impairment, sleep disturbances, and autonomic dysfunction (Hayes, 2019; Morris et al., 2024). With a global prevalence exceeding six million cases, PD ranks as the second most common neurodegenerative disorder after AD, imposing substantial socioeconomic burdens (Ascherio and Schwarzschild, 2016; Weintraub et al., 2022). The etiology of PD remains multifactorial, involving complex interactions between genetic predisposition, environmental exposures, and aging-related cellular dysfunction (Lotankar et al., 2017; Tysnes and Storstein, 2017). Mutations in genes encoding α-synuclein, leucine-rich repeat kinase 2, and PARKIN have provided crucial insights into pathogenic mechanisms, including mitochondrial dysfunction, proteostasis failure, and neuroinflammation (Jankovic and Tan, 2020; Brooker and Gonzalez-Latapi, 2025; Xiao et al., 2025). Current therapeutic approaches primarily focus on dopamine replacement using levodopa, which provides symptomatic relief but fails to halt disease progression. Emerging disease-modifying strategies targeting α-synuclein pathology or employing gene-based interventions show promise but require further validation (Du et al., 2020; Chen et al., 2022). Curcumin, a natural polyphenol, demonstrates significant potential in PD and other neurodegenerative conditions. Experimental evidence indicates that curcumin exerts neuroprotective effects by modulating the BDNF/PI3k/Akt signaling pathway, which plays a critical role in neuroregeneration and anti-apoptotic processes (Jin et al., 2022). Furthermore, curcumin prevents rotenone-induced PD in murine models by inhibiting microglial NLRP3 inflammasome activation and mitigating mitochondrial dysfunction (Xu et al., 2023). In rotenone-exposed mice, curcumin administration activates the p62-Keap1-Nrf2 pathway, enhances autophagy, and improves antioxidant capacity (Rathore et al., 2023). Additional mechanisms include neuroprotection through HDAC6-NLRP3 pathway modulation and amelioration of MPTP-induced gastrointestinal dysfunction, gut microbiota dysbiosis, and short-chain fatty acid profile alterations (Cai et al., 2025). To enhance therapeutic efficacy, advanced delivery systems have been developed, including mitochondria-targeting biomimetic nanoparticles functionalized with curcumin (Zhu et al., 2022; Cai et al., 2023). These nanoparticles localize to damaged neuronal mitochondria in inflammatory environments and modulate the NAD+/SIRT1/PGC-1α/PPARγ/NRF1/TFAM signaling cascade, alleviating MPP + -induced neuronal toxicity and mitochondrial dysfunction (Zheng et al., 2023). Moreover, curcumin-loaded nanoemulsions have demonstrated superior efficacy in preventing motor deficits and inhibiting complex I dysfunction, representing promising nanomedicine applications for PD (Ramires Junior et al., 2021). While preclinical studies consistently demonstrate the ability of curcumin to mitigate PD symptoms and attenuate neuroinflammation *in vivo*, rigorous clinical trials remain necessary to substantiate its therapeutic potential for patients with PD and establish optimal treatment protocols.

### 4.3 Multiple sclerosis (MS)

MS is a chronic autoimmune-mediated demyelinating disease of the central nervous system, characterized by multifocal inflammatory lesions, axonal damage, and progressive neurological dysfunction (Kuhlmann et al., 2023). As the most prevalent non-traumatic cause of neurological impairment in young adults, MS affects approximately 2.8 million individuals worldwide, with a higher prevalence among females and in temperate geographical regions. The clinical manifestations of MS exhibit significant heterogeneity, ranging from relapsing-remitting episodes to progressive neurological decline, reflecting a complex interplay between genetic susceptibility and environmental triggers (Travers et al., 2022). The pathogenesis of MS involves the infiltration of autoreactive T cells across the blood-brain barrier, initiating an inflammatory cascade targeting myelin, followed by oligodendrocyte loss and impaired axonal conduction (Perez et al., 2023). Although the precise etiology remains elusive, genome-wide association studies have identified over 200 risk loci, particularly within the major histocompatibility complex region, underscoring the importance of immune dysregulation (Hauser and Cree, 2020). Current therapeutic options for the disease, such as anti-CD20 monoclonal antibodies and sphingosine-1-phosphate receptor modulators, demonstrate limited efficacy in progressive forms of MS (de Seze et al., 2023; Klotz et al., 2023). Th17 cells are critical immune participants in the pathophysiology of MS (Qureshi et al., 2018). Curcumin inhibits the proliferation of Th17 cells and reduces the production of pro-inflammatory cytokines, including TNF-α, IL-22, and IL-17, offering therapeutic potential for MS (Ghoushi et al., 2024). In addition, curcumin exerts neuroprotective effects by downregulating AXL-mediated astrocyte-driven inflammation in cuprizone-induced mouse models, targeting MS treatment (Zhang et al., 2025). In EAE mouse models and LPS-stimulated BV-2 microglial cells, curcumin may ameliorate microglial-mediated inflammatory responses by inhibiting the AXL/JAK2/STAT3 signaling pathway (Sun et al., 2022). Reports indicate that curcumin and its semi-synthetic derivative F-curcumin suppress the expression of IL-1β, IL-4, IL-10, IL-17, interferon-γ, and TGF-β during EAE induction, mitigating MS-associated inflammation (Khosropour et al., 2023). To enhance the bioavailability of curcumin in the treatment of MS, researchers have designed a prodrug, curcumin monoglucuronide, which, when administered intravenously or intraperitoneally, alters the overall gut microbiome composition and modifies the abundance of specific bacterial populations to suppress neuroinflammation and improve MS outcomes (Khadka et al., 2021). In materials science, investigators have utilized high-density lipoprotein-mimicking peptide-phospholipid scaffolds (HPPS) as carriers to improve the bioavailability of curcumin, effectively reducing inflammatory monocyte infiltration across the blood-brain barrier, inhibiting microglial proliferation, and limiting the infiltration of other effector immune cells, thereby decreasing the incidence of EAE in mice (Lu et al., 2020). Similarly, polymeric forms of nano-curcumin correct the balance of pro-inflammatory and anti-inflammatory gene expression in EAE models, reduce oxidative stress, enhance myelin regeneration, and increase progenitor cell markers (Mohajeri et al., 2015).

In summary, curcumin demonstrates the potential to inhibit neuroinflammation in EAE models, improving outcomes in MS.

### 4.4 Stroke

Stroke represents a significant global health burden, ranking as the second leading cause of death and the third leading cause of disability worldwide. This cerebrovascular event occurs when arterial occlusion or vessel rupture interrupts blood flow to the brain, resulting in the rapid onset of neurological deficits (Kong et al., 2022; Hilkens et al., 2024). The pathophysiological cascade involves excitotoxicity, oxidative stress, and neuroinflammation, ultimately leading to neuronal death within minutes to hours following ischemic injury (Feigin et al., 2025). Recent advancements in the management of acute stroke, particularly the expansion of thrombolytic time windows and the widespread adoption of endovascular thrombectomy, have fundamentally altered the treatment landscape for ischemic stroke (Bushnell et al., 2024). However, significant challenges persist, including narrow therapeutic time windows, the risk of hemorrhagic transformation, and limited neuroprotective strategies (Caso et al., 2024). Moreover, recovery post-stroke remains suboptimal for many patients, with approximately 50% of survivors experiencing long-term disabilities. Therefore, identifying more effective therapeutic targets is critical for enhancing stroke rehabilitation (Kong et al., 2024a; Wegener et al., 2024). Research indicates that curcumin partially mitigates stroke-induced white matter injury and improves neurological function by inhibiting the NF-κB/NLRP3 signaling pathway, providing neuroprotection following a stroke (Ran et al., 2021). Furthermore, curcumin pretreatment enhances ischemic stroke outcomes by preserving blood-brain barrier integrity, promoting synaptic remodeling, and downregulating the phosphorylation of NF-κB and MMP-9, thereby suppressing inflammatory responses (Wu et al., 2021). In models of intracerebral hemorrhage (ICH), curcumin treatment facilitates the inhibition of oxidative stress in microglia by activating the Nrf2/HO-1 pathway and promoting neurological recovery post-ICH, thereby alleviating neuronal damage (Duan et al., 2022). To enhance the therapeutic efficacy of curcumin in stroke, a combined therapy utilizing curcumin and human umbilical cord-derived mesenchymal stem cells exerts anti-inflammatory and antioxidant effects through the AKT/GSK-3β/β-TrCP/Nrf2 axis, improving neurological function following acute ischemic stroke (Li et al., 2023). Similarly, in materials science, researchers have encapsulated curcumin in mPEG-PCL nanoparticles, which are administered intranasally to deliver curcumin directly to the brain. This approach reprograms pro-inflammatory microglia to an anti-inflammatory phenotype, reducing neuronal inflammatory death and hematoma volume in mouse models of ICH (Duan et al., 2024). Additionally, encapsulating curcumin in polymer-based nanoparticles has shown superior therapeutic effects compared with curcumin alone in inhibiting erastin-induced ferroptosis in HT22 hippocampal cells (Yang et al., 2021a).

In summary, curcumin exhibits neuroprotective properties in ischemic and hemorrhagic stroke models by inhibiting neuroinflammation and mitigating neuronal damage, thereby promoting recovery from stroke.

### 4.5 Amyotrophic lateral sclerosis (ALS)

ALS is a devastating neurodegenerative disorder characterized by the progressive degeneration of upper and lower motor neurons, leading to muscle weakness, paralysis, and ultimately respiratory failure within 3–5 years of symptom onset (Feldman et al., 2022). The global prevalence of ALS is approximately 4–6 cases per 100,000 individuals, making it the most common motor neuron disease among adults and imposing significant physiological and psychological burdens on patients and caregivers (Goutman et al., 2022; Akcimen et al., 2023). The pathogenesis of ALS involves a complex interplay between genetic susceptibility, particularly mutations in C9ORF72, superoxide dismutase 1 (SOD1), TARDBP, and fused in sarcoma (FUS), and environmental factors, resulting in multiple pathological mechanisms, including protein misfolding, oxidative stress, mitochondrial dysfunction, and neuroinflammation. Despite extensive research efforts, current therapeutic options remain limited (Goutman et al., 2022; Ilieva et al., 2023).

Recent studies have identified solid lipid curcumin particles as a potential estrogen replacement therapy that may mitigate the progression and pathogenesis of TDP-43-related diseases (Majumder et al., 2025). In addition, curcumin treatment enhances ATP levels by attenuating the sequestration of pyruvate kinase mediated by FUS aggregation, thereby offering a promising avenue for ALS treatment (Shi et al., 2023). Furthermore, researchers have discovered a novel potent polyphenolic compound, ethoxycurcumin, an effective inhibitor in reducing the risk of fatal ALS by preventing the abnormal misfolding and aggregation of SOD1 into amyloid aggregates (Kouhi et al., 2024). This mechanism may be due to the stronger binding affinity of curcumin to SOD1 protofibrils, facilitated by greater van der Waals interactions (Sharma et al., 2023). Collectively, these findings indicate that curcumin significantly inhibits ALS symptoms *in vitro* and *in vivo*, providing new therapeutic directions for improving the prognosis of ALS.

### 4.6 Epilepsy and seizures

Epilepsy is a chronic neurological disorder characterized by recurrent, unprovoked seizures resulting from abnormal, synchronous neuronal activity in the brain. It is among the most prevalent neurological conditions, significantly impacting morbidity, mortality, and quality of life (Specchio et al., 2022). Seizures can manifest in various clinical phenotypes, ranging from brief focal awareness seizures to generalized convulsive events, reflecting underlying network dysfunction (Deng and Husari, 2024; Krishnamurthy, 2025). The mechanisms underlying epilepsy include ion channel dysfunction (such as SCN1A mutations in Dravet syndrome), heightened excitability of glutamatergic pathways, and impaired GABAergic inhibition. Despite the availability of antiepileptic drugs, approximately 30% of patients experience drug-resistant epilepsy, necessitating alternative interventions such as surgical resection, neurostimulation, or dietary therapies. Consequently, novel therapeutic approaches are essential (Kanner et al., 2024; Pellinen et al., 2024). Recent studies have demonstrated that curcumin can significantly reduce the frequency of seizures in the clinical treatment of pediatric refractory epilepsy (Erfani et al., 2022). In rat models of epilepsy, curcumin administration markedly decreased seizure-like activity, with reduced mRNA and protein levels of Na+, thereby diminishing seizure occurrences (Kumar et al., 2019). In a pentylenetetrazol (PTZ)-induced seizure model, curcumin exerted anticonvulsant effects by elevating serotonin levels in the brain, influencing receptors such as 5-HT1A, 5-HT2C, and 5-HT4, and potentially by downregulating 5-HT7 gene expression (Arbabi Jahan et al., 2018). Moreover, curcumin attenuates glial cell activation and ameliorates cognitive deficits in patients with chronic epilepsy (Kaur et al., 2015). In a lithium-pilocarpine rat model inducing status epilepticus, positron emission tomography revealed that curcumin treatment inhibited cerebral glucose metabolism, reduced body weight, mitigated hippocampal neuronal damage, and decreased neuroinflammation, ultimately reducing seizure frequency (Slowing et al., 2023). Furthermore, in the same model, curcumin conferred neuroprotection by inducing autophagy and inhibiting necroptotic apoptosis, safeguarding hippocampal neurons from status epilepticus-induced injury (Wang et al., 2017). To enhance the anticonvulsant properties of curcumin and improve its bioavailability, researchers have micronized the compound using supercritical carbon dioxide processing. In adult zebrafish models of PTZ-induced seizures, micronized curcumin exhibited effects comparable to those of classical antiepileptic drugs (Bertoncello et al., 2018).

**FIGURE 3:**
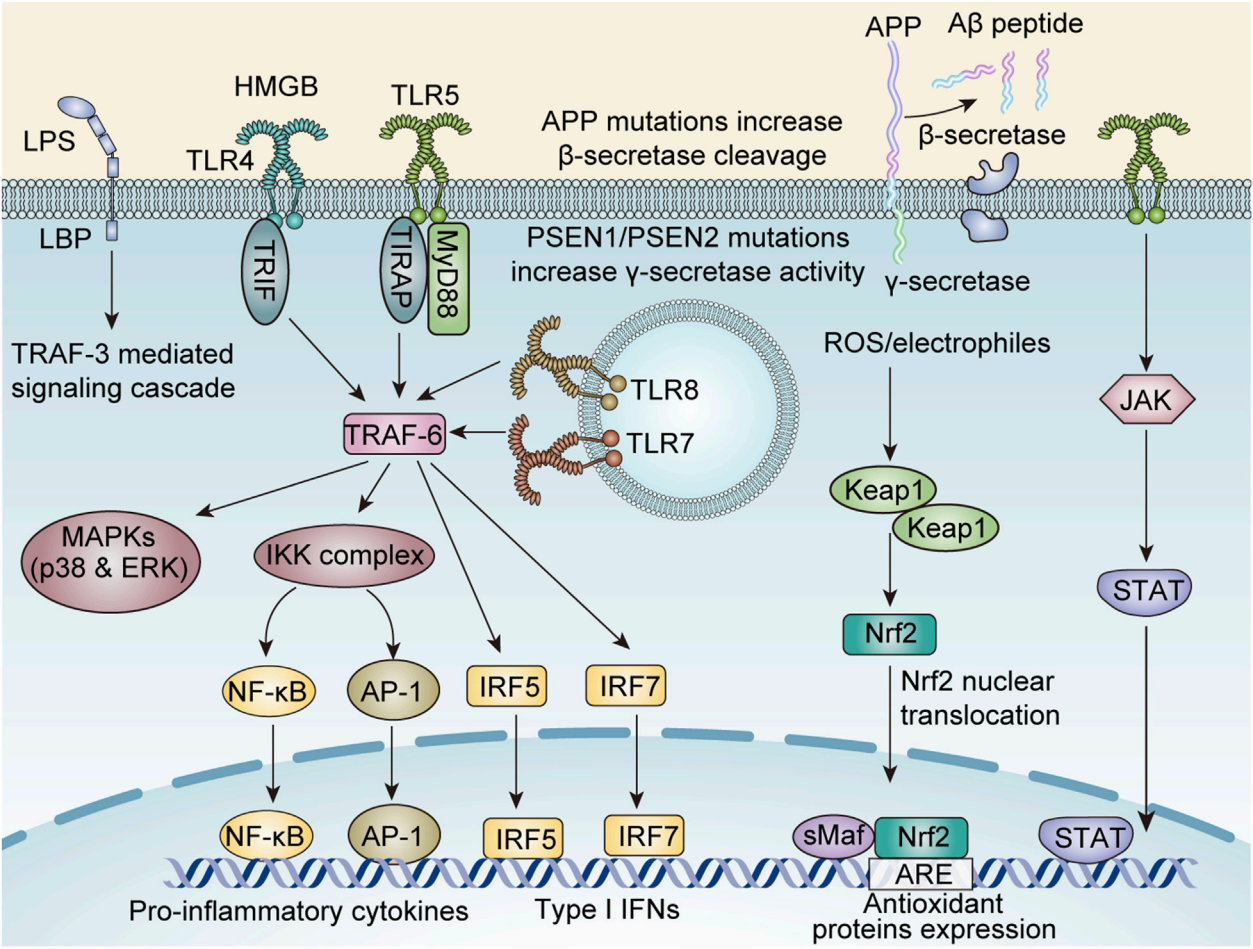
The molecular mechanisms underlying curcumin-mediated anti-neuroinflammatory effects involve multiple signaling pathways, including: the TNF receptor associated factors 6 (TRAF-6) -mediated nuclear factor kappa-B (NF-κB), activator protein-1 (AP-1), Interferon Regulatory Factor 5 (IRF5), IRF7, and mitogen-activated protein kinase (MAPK)/p38/Extracellular regulated protein kinases (ERK) axis; the Janus Kinase (JAK)/signal transducer and activator of transcription (STAT) axis; the reactive oxygen species (ROS)-Kelch-like ECH-associated protein 1 (KEAP1)/nuclear factor erythroid 2-related factor 2 (Nrf2) axis; the TRAF-3 axis; and amyloid-beta (Aβ) protein modulation.

In summary, curcumin demonstrates potential as an antiepileptic agent in various models by suppressing neuroinflammation, thereby reducing seizure frequency (Figure 3).

## 5 Discussion

**FIGURE 4:**
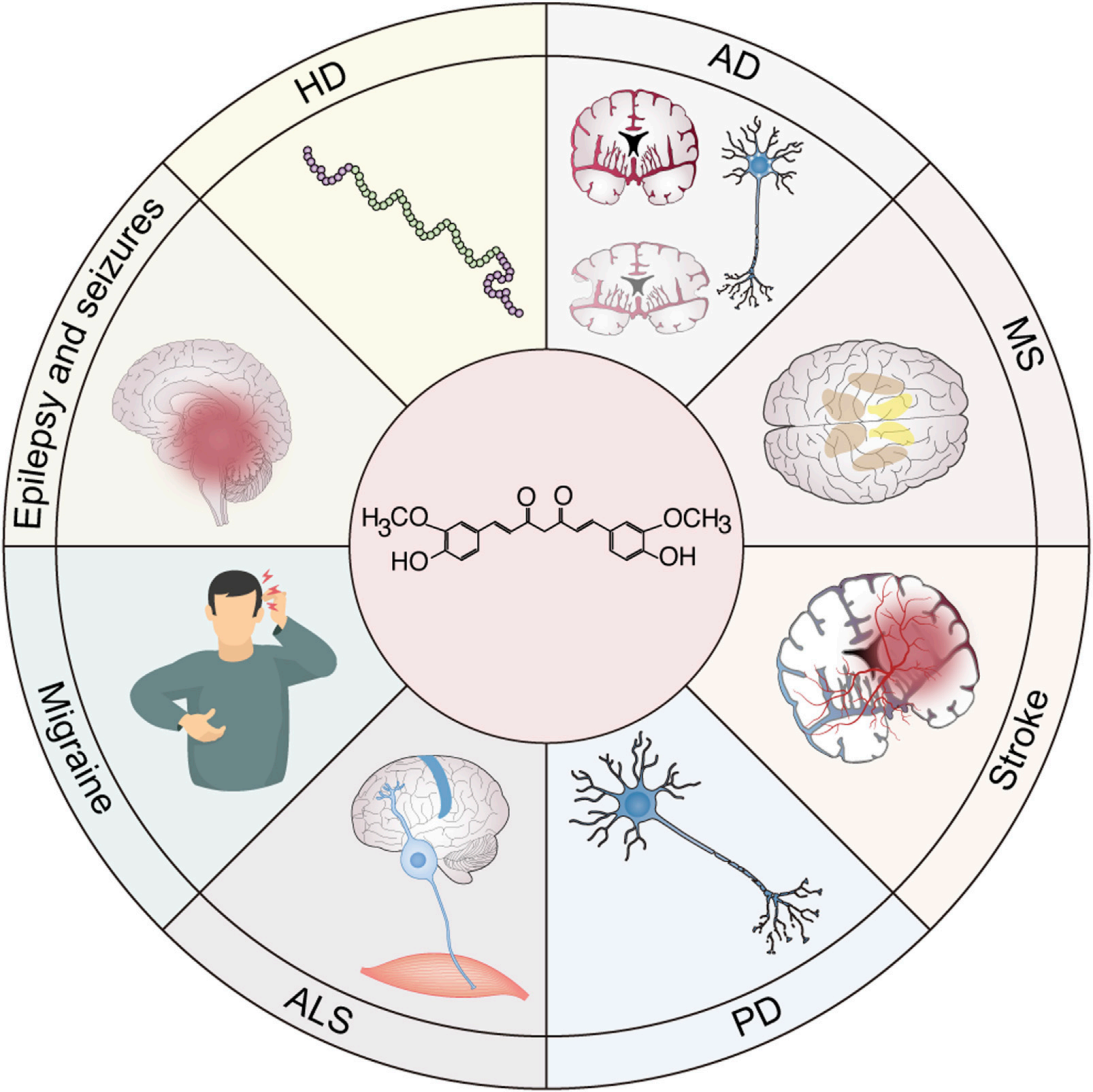
Potential application of curcumin in neuroinflammatory diseases. Neuroinflammation is a common pathogenic mechanism of various neurological diseases, including stroke, multiple sclerosis (MS), Parkinson’s disease (PD), Alzheimer’s disease (AD), Huntington’s disease (HD), epilepsy and seizures, migraine, and amyotrophic lateral sclerosis (ALS). Therefore, curcumin shows great potential as a prodrug in the clinical translation of these inflammation-related neurological diseases.

Neurodegenerative diseases (NDs), including stroke, AD, PD, ALS, and Huntington’s disease, are increasingly recognized as complex multifactorial disorders characterized by the interplay of chronic neuroinflammation, oxidative stress, and progressive neuronal degeneration (Polissidis et al., 2020; Gupta et al., 2023; Kong et al., 2023; Zhou et al., 2023; Goetzl, 2025). A growing body of evidence underscores the pivotal role of sustained neuroinflammatory processes in the onset and progression of these debilitating diseases. The neuroinflammatory cascade in NDs is primarily mediated by the persistent activation of resident immune cells in the central nervous system, namely microglia and astrocytes (Agnello and Ciaccio, 2022; Teleanu et al., 2022). This pathological activation initiates a self-perpetuating inflammatory cycle characterized by the excessive production of pro-inflammatory cytokines (such as TNF-α, IL-1β, and IL-6), ROS, and neurotoxic mediators (Youwakim and Girouard, 2021; Gao et al., 2023). Notably, the NLRP3 inflammasome has emerged as a critical molecular platform linking neuroinflammation and neurodegeneration, facilitating the maturation and secretion of IL-1β and IL-18 in response to pathological protein aggregation (Coll et al., 2022). Recent advancements in neuroimmunology indicate that the neuroinflammatory process exhibits neuroprotective and neurotoxic effects, depending on the disease stage and microenvironment (Nainu et al., 2023). While acute inflammation can promote tissue repair and pathogen clearance, chronic inflammation drives progressive neurodegeneration through feedforward loops involving damage-associated molecular patterns and sustained immune activation (Castro-Gomez and Heneka, 2024; Yu et al., 2025). Understanding these complex neuroimmune interactions provides crucial insights for developing targeted therapeutic strategies for modulating rather than suppressing neuroinflammatory responses (Kong et al., 2024c). Current research efforts are focused on identifying key regulatory nodes within the neuroinflammatory cascade that can serve as therapeutic targets, potentially disrupting the cycle of inflammation-mediated NDs (Figure 4).

The pharmacological treatment strategies for NDs approved by regulatory agencies such as the Food and Drug Administration and European Medicines Agency are primarily palliative, focusing on symptom management rather than addressing the underlying neuropathological mechanisms (Zhang et al., 2023c; Cantara et al., 2024). These conventional approaches, including acetylcholinesterase inhibitors for AD and dopaminergic replacement therapies for PD, are often associated with significant adverse effects and demonstrate limited efficacy in halting disease progression (Grayson, 2016; Beata et al., 2023). Furthermore, these therapies do not modulate the chronic neuroinflammatory processes that are increasingly recognized as key drivers of neurodegeneration (Zhang et al., 2023c). In contrast, extensive preclinical research has identified curcumin and its derivatives as multimodal neuroprotective agents that can target the fundamental inflammatory pathways associated with NDs (Lo Cascio et al., 2021; Genchi et al., 2024). Mechanistic studies indicate that curcumin exerts its therapeutic effects through the complex modulation of various neuroinflammatory signaling cascades, including the NF-κB pathway, NLRP3 inflammasome activation, and Nrf2-mediated antioxidant responses, while downregulating pro-inflammatory cytokines (such as IL-6 and TNF-α) and upregulating anti-inflammatory mediators (such as TGF-β and IL-10) (Zia et al., 2021; Erfani et al., 2022; Tripathi and Bhawana, 2024). Curcumin possesses the ability to simultaneously modulate multiple signaling pathways, conferring a comprehensive advantage compared to many single-target synthetic drugs or other flavonoids such as quercetin and resveratrol (Akaberi et al., 2021). Notably, curcumin can activate the BDNF/TrkB pathway, thereby promoting synaptic growth and hippocampal neurogenesis—a feature rarely observed in most synthetic drugs (e.g., acetylcholinesterase inhibitors) and superior to that of some flavonoids which only exhibit anti-inflammatory or antioxidant properties (Yang et al., 2020). Furthermore, curcumin is naturally low in toxicity, and long-term use is associated with significantly fewer side effects than synthetic drugs such as nonsteroidal anti-inflammatory drugs or immunosuppressants. While other flavonoids like resveratrol may impose hepatic or renal burden at high doses, curcumin has a well-established safety profile at appropriate dosages (Pourbagher-Shahri et al., 2021). Curcumin may offer a more favorable safety profile compared to the broad immunosuppressive effects associated with many conventional anti-inflammatory drugs.

Despite its compelling therapeutic potential, the clinical translation of curcumin is severely hampered by a constellation of pharmacokinetic limitations. Most prominent among these is its exceedingly low systemic bioavailability, which profoundly restricts its therapeutic efficacy. This deficiency arises from a combination of factors including poor aqueous solubility, inadequate absorption from the gastrointestinal tract, rapid metabolic inactivation, and swift systemic elimination. Consequently, even after oral administration of high doses, plasma and tissue concentrations of the native compound remain substantially below the levels required to elicit significant pharmacological effects within the central nervous system. Recent advancements in nanotechnology-based drug delivery systems have significantly transformed the therapeutic potential of curcumin in bioavailability pathways associated with NDs (Liu et al., 2024b; Mohammadzadeh et al., 2024). Nanostructured curcumin demonstrates considerable potential in reducing therapeutic doses. Through nanotechnology-based formulation, the particle size of curcumin can be effectively reduced, while its surface charge and specific surface area are optimized. Moreover, such processing facilitates the formation of a high-energy amorphous state stabilized by intermolecular hydrogen bonding (Mohammadzadeh et al., 2024). Compared to free curcumin, its nanoformulations exhibit not only significantly improved aqueous solubility and drug release profiles but also enhanced antioxidant and antitumor activities (Ratan et al., 2023). Furthermore, nano-carrier systems can effectively shield the drug from detrimental environmental factors, markedly improving its physicochemical stability. Owing to these multiple advantages—including superior stability, protection of the drug, controlled release properties, prolonged *in vivo* circulation time, and efficient drug loading without the need for chemical modification—both synthetic and natural polymers have been extensively employed to develop curcumin nano-delivery systems (Hajimirzaei et al., 2025). Commonly used polymeric carriers include poly (lactic-co-glycolic acid) (PLGA), polycaprolactone (PCL), poly (N-isopropylacrylamide) (PNIPAAm), chitosan, dextrin, polyethylene glycol (PEG), and polyvinyl alcohol (PVA). Various preparation techniques—such as nanoprecipitation, high-pressure homogenization, emulsion-solvent evaporation, and chemical crosslinking—have been utilized to fabricate these nanoparticles (Del Prado-Audelo et al., 2019). These methods enable efficient encapsulation of curcumin into polysaccharide-based nanoparticles, thereby significantly enhancing its stability and enabling controlled drug release. Sophisticated formulations, including nanoparticles, solid lipid nanoparticles, and liposomal carriers, have achieved notable success in enhancing the pharmacokinetic properties of curcumin through various mechanisms (Mahjoob and Stochaj, 2021). These enhancements include improved bioavailability, increased permeability across the blood-brain barrier, and targeted anti-inflammatory effects (Yang et al., 2024b; Hajimirzaei et al., 2025). Such innovative technologies have augmented the therapeutic potential of curcumin in neuroinflammation-related conditions, demonstrating the ability to reverse cognitive deficits, reduce oxidative stress markers, and maintain synaptic density (Attaluri et al., 2022; Ghaffari et al., 2024; Yang et al., 2024b).

However, the clinical translation of these findings remains constrained by the limitations of human studies and ethical considerations. Based on the current available evidence, curcumin demonstrates considerable potential in modulating neuroinflammation. Nevertheless, translating this promise into clinical applications requires addressing several critical challenges, including but not limited to: conducting large-scale, randomized controlled trials with rigorously defined endpoints, validating mechanistic pathways in human subjects, standardizing bioavailable formulations, and exploring adjunctive and combination therapies. Consequently, more extensive and well-designed trials are imperative to establish optimal dosing regimens and long-term safety profiles.

## 6 Conclusion

In conclusion, while curcumin exhibits promising therapeutic value in the context of neuroinflammation-related diseases, its efficacy and prognostic outcomes remain inconsistent. Consequently, it is imperative to develop and optimize curcumin treatment protocols—including administration routes and dosing strategies—to enhance its clinical effectiveness.

## Glossary

NF-κB: Nuclear factor kappa-B
NLRP3: NOD-like receptor thermal protein domain associated protein 3
Nrf2: Nuclear Factor Erythroid 2-related Factor 2
TGF-β: Transforming growth factor β
IL-10: Interleukin 10
CNS: Central nervous system
TNF-α: Tumor necrosis factor-α
IL-1β: Interleukin-1 β
IL-6: Interleukin- 6
ROS: Reactive oxygen species
AD: Alzheimer’s disease
PD: Parkinson’s disease
MS: Multiple sclerosis
TLRs: Toll-like receptors
BBB: Blood-brain barrier
NSAIDs: Non-steroidal anti-inflammatory drugs
MAPK: Mitogen-Activated Protein Kinase
PI3K/Akt: Phosphatidylinositol 3-Kinase/Akt
HAT: Hydrogen atom transfer
SET: Single electron transfer
STAT3: Signal transducer and activator of transcription 3
LPS: Lipopolysaccharide
NO: Nitric oxide
HO-1: Heme Oxygenase-1
TBI: Traumatic brain injury
SAH: Subarachnoid hemorrhage
TREM2: Triggering receptor expressed on myeloid cells 2
EAE: Experimental autoimmune encephalomyelitis
Aβ: Amyloid-β
AChEIs: Acetylcholinesterase inhibitors
DG: Dentate gyrus
T2D: Type 2 diabetes
GBE: Ginkgo biloba extract
P-tau: Phosphorylated tau
MDA: Malondialdehyde
GSH: Glutathione
CNPs: Curcumin nanoparticles
SNCA: α-synuclein
LRRK2: Leucine-rich repeat kinase 2
MHC: Major histocompatibility complex
CPZ: Cuprizone
IFN-γ: Interferon-γ
HPPS: High-density lipoprotein-mimicking peptide-phospholipid scaffold
PNC: Polymeric forms of nano-curcumin
hUC-MSC: Human umbilical cord-derived mesenchymal stem cells
ALS: Amyotrophic lateral sclerosis
SLCP: Solid lipid curcumin particles
FUS: Fused in sarcoma
SOD1: Superoxide dismutase 1
PTZ: Pentylenetetrazol
PET: Positron emission tomography
NDs: Neurodegenerative diseases
HD: Huntington’s disease
DAMPs: Damage-associated molecular patterns
NPs: Nanoparticles
SLNs: Solid lipid nanoparticles
